# Monoclonal Gammopathy of Renal Significance in a Patient With Cryoglobulinemia and Nephrotic Syndrome: A Case Report

**DOI:** 10.7759/cureus.86043

**Published:** 2025-06-15

**Authors:** Faisal Aleisa, Mutrik Almughni, Essam Jameel, Nourah Al Oudah, Junaid Iqbal

**Affiliations:** 1 College of Medicine, King Saud Bin Abdulaziz University for Health Sciences, Riyadh, SAU; 2 Department of Pathology and Laboratory Medicine, King Abdulaziz Medical City, Ministry of National Guard Health Affairs (MNGHA), Riyadh, SAU; 3 Department of Medicine/Division of Nephrology, King Abdulaziz Medical City, Ministry of National Guard Health Affairs (MNGHA), Riyadh, SAU

**Keywords:** cryoglobulinemia, igm kappa, membranoproliferative glomerulonephritis (mpgn), monoclonal gammopathy of renal significance (mgrs), nephrotic syndrome

## Abstract

We report a case of monoclonal gammopathy of renal significance (MGRS) in a 65-year-old female patient who presented to the emergency department with uncontrolled hypertension, generalized edema, abdominal distension, and frothy urine. Apart from recently diagnosed hypertension, she had no significant comorbidities. Examination revealed periorbital swelling, bibasal crackles, moderate ascites, and bilateral lower limb pitting edema. Laboratory findings showed proteinuria, low complement levels, elevated IgM, decreased IgG, and the presence of cryoglobulins. Imaging and PET-CT ruled out malignancy. Kidney biopsy revealed membranoproliferative glomerulonephritis with cryoglobulin deposition and vasculitis. Bone marrow biopsy confirmed plasma cell dyscrasia with kappa light chain restriction. Despite suspicion of Waldenström macroglobulinemia or IgM myeloma, cytogenetics and fluorescence in situ hybridization were negative. The patient was treated with rituximab and corticosteroids, with complete resolution of ascites, edema, and proteinuria. This case highlights an uncommon presentation of IgM kappa-related MGRS with type I cryoglobulinemia.

## Introduction

Monoclonal gammopathy of undetermined significance (MGUS) is a clinically silent premalignant plasma cell disorder that is characterized by the presence of M-proteins less than 30 g/L in the serum and clonal plasma cells fewer than 10% in the bone marrow and no evidence of organ damage or any other disorder that explains it [1]. When renal involvement is present, the condition is then classified as monoclonal gammopathy of renal significance (MGRS), a separate diagnosis that requires early recognition and treatment due to its potential to cause irreversible kidney damage [2].

Cryoglobulinemia is a rare condition characterized by immunoglobulins that precipitate at cold temperatures and can result from various etiologies such as systemic autoimmune diseases, malignant neoplasms, and chronic infections, and in some cases, have no identified etiology (essential cryoglobulinemia) [3]. Furthermore, while cryoglobulinemia may lead to various forms of glomerulonephritis, its presentation as nephrotic syndrome is uncommon, particularly in the context of MGRS [3].

We present a case of MGRS in a 65-year-old female patient presenting with nephrotic syndrome due to IgM monoclonal gammopathy leading to cryoglobulinemia and membranoproliferative glomerulonephritis (MPGN).

## Case presentation

A 65-year-old female patient was recently noted to have elevated blood pressure in the months preceding her presentation to the emergency department with generalized swelling, abdominal distension, and uncontrolled hypertension. The patient reported a one-month history of progressively worsening generalized edema and frothy urine.

Apart from recently diagnosed hypertension, she had no significant comorbidities nor any history of recent intercurrent illness. On examination, she had peri-orbital swelling, appeared mildly short of breath at rest, had a blood pressure of 189/91 mmHg but was not tachypneic or tachycardic and maintaining oxygen saturation of 99% on room air. Her chest auscultation revealed bibasal crackles and she had moderate ascites on abdominal examination. Her cardiac examination was unremarkable, and she had no visible rash. She had a bilateral lower limb pitting edema up to her knees. There was noticeable swelling of the left breast compared to the right, with warmth and erythema, but it was not tender, and she had no accessible palpable lymphadenopathy. She was investigated for the above outlined clinical findings and to evaluate the etiology of nephrotic syndrome. Her initial lab results are summarized in Table 1.

**Table 1:** Laboratory evaluation. MCHC: Mean corpuscular hemoglobin concentration; MCH: Mean corpuscular hemoglobin; RDW: Red cell distribution width; MPV: Mean platelet volume

Lab results	Patient values	Reference range
Hemoglobin	90 gm/L	120 – 160 gm/L
White blood cell count	7.60 x10^9/L	4 ~ 11x10^9/L
Red blood cell count	3.70 x10^12/L	4~5.4 x10^12/L
Hematocrit	0.286 L/L	0.36 ~ 0.54 L/L
MCV	77.2 fL	76 – 96 fL
MCH	24.4 pg	27- 32 pg
MCHC	316 g/L	320 – 350 g/L
RDW	15.3 %	11.5-14.5%
Platelet	273x10^9/L	150-400x10^9/L
MPV	7.7 fL	6.3-10.3fL
Sodium	135 mmol/L	136 -145 mmol/L
Potassium	3.8 mmol/L	3.5 – 5.1 mmol/L
Chloride	100 mmol/L	98-107 mmol/L
Blood urea nitrogen	7.9 mmol/L	3.5 – 7.2 mmol/L
Bicarbonate	26 mmol/L	23 – 31 mmol/L
Creatinine	75 umol/L	50 – 98 umol/L
Estimated glomerular filtration rate	71 mL/min/1.73m^2^	60 ~ mL/min/1.73m^2^
Random blood glucose	6.4 mmol/L	2.9 – 7.8 mmol/L
Creatine kinase	32 U/L	29 – 168 U/L
Adjusted Calcium	2.06 mmol/L	2.2 – 2.5 mmol/L
Total bilirubin	6.1 umol/L	~ 20.5 umol/L
Albumin	27 g/L	32 – 46 g/L
Total protein	58 g/L	64 – 83 g/L
Aspartate aminotransferase	24 U/L	5 – 34 U/L
Alanine aminotransferase	22 U/L	5 – 55 U/L
Alkaline phosphatase	210 U/L	40 – 150 U/L
Glycated hemoglobin	5.5%	<5.7%
Urinalysis		
Color	Yellow	Pale yellow-dark yellow
Appearance	Slightly cloudy	Clear
Specific gravity	1.014	1.015-1.030
pH	5.0	5-7
Urine glucose	50 mg/dL	Negative
Urine ketones	Negative	Negative
Urine blood	0.10	Negative
Urine leukocyte esterase	Negative	Negative
Urine nitrite	Negative	Negative
Urine bilirubin	Negative	Negative
Urine urobilinogen	Normal	Trace
Urine protein	100 mg/dL	Negative
Urine Red blood cells	9/hpf	0-5/hpf
Urine white blood cells	6/hpf	0-5/hpf
Urine mucous	+	None
Urine hyaline cast	>30/hpf	None
Urine Protein/Creatinine Ratio	6.82 g/g	0.2 g/g
24-hour urine protein	5.2 gm/day	0.3gm/day
Complement (C3) levels	0.535 g/L	0.79-1.52 g/L
Complement (C4) levels	0.022 g/L	0.160 – 0.380 g/L
Antistreptolysin O antibodies	40 KIU/L	~116 KIU/L
Immunoglobulin A serum	1.81 g/L	0.82 – 4.53 g/L
Immunoglobulin G serum	6.82 g/L	7.51- 15.60 g/L
Immunoglobulin M serum	11.70 g/L	0.46 – 3.04 g/L
Serum free kappa light chains	360 mg/L	~19.40 mg/L
Serum free lambda light chains	28.30 mg/L	5.71- 26.30 mg/l
Free kappa/lambda ratio	12.72	0.26 – 1.65
Cryoglobulins	Present	
International normalized ratio	0.93	0.80 – 1.20
Prothrombin time	10.20 seconds	9.38-12.34 seconds
Partial thromboplastin time	23.60 seconds	24.84-32.96 seconds
Hepatitis B surface antigen	Non-reactive	Non-reactive
Hepatitis C antibody	Non-reactive	Non-reactive
Malaria smear	None seen	None seen
Tuberculosis	Indeterminate	Negative
Epstein-Barr Nuclear Antigen Immunoglobulin G	<3 U/ML	<5 U/ML
Epstein-Barr Virus Early Antigen	32.30 U/ML	<10 U/ML negative 10-40 U/ML equivocal >Or =40 U/ML positive
Varicella Zoster Virus Immunoglobulin G	62.16 mIU/ml	<150 mIU/ml
Anti-nuclear antibody	7.11 unit	<20 unit
Anti-double-stranded DNA	6.58 IU/mL	<200 IU/mL
Anti-phospholipase A2 receptor antibodies	<1:10	<1:10
Glomerular basement membrane antibodies	0.26 unit	0-20 unit
Myeloperoxidase antibodies	1.61 unit	<20 unit
Proteinase-3 antibodies	1.04 unit	<20 unit
Rheumatoid factor	65.13 IU/mL	~30 IU/mL
Inflammatory markers		
Erythrocyte sedimentation rate	85 mm/hr	0-30 mm/hr
​C-reactive protein	16 mg/L	~8 mg/L

**Figure 1:**
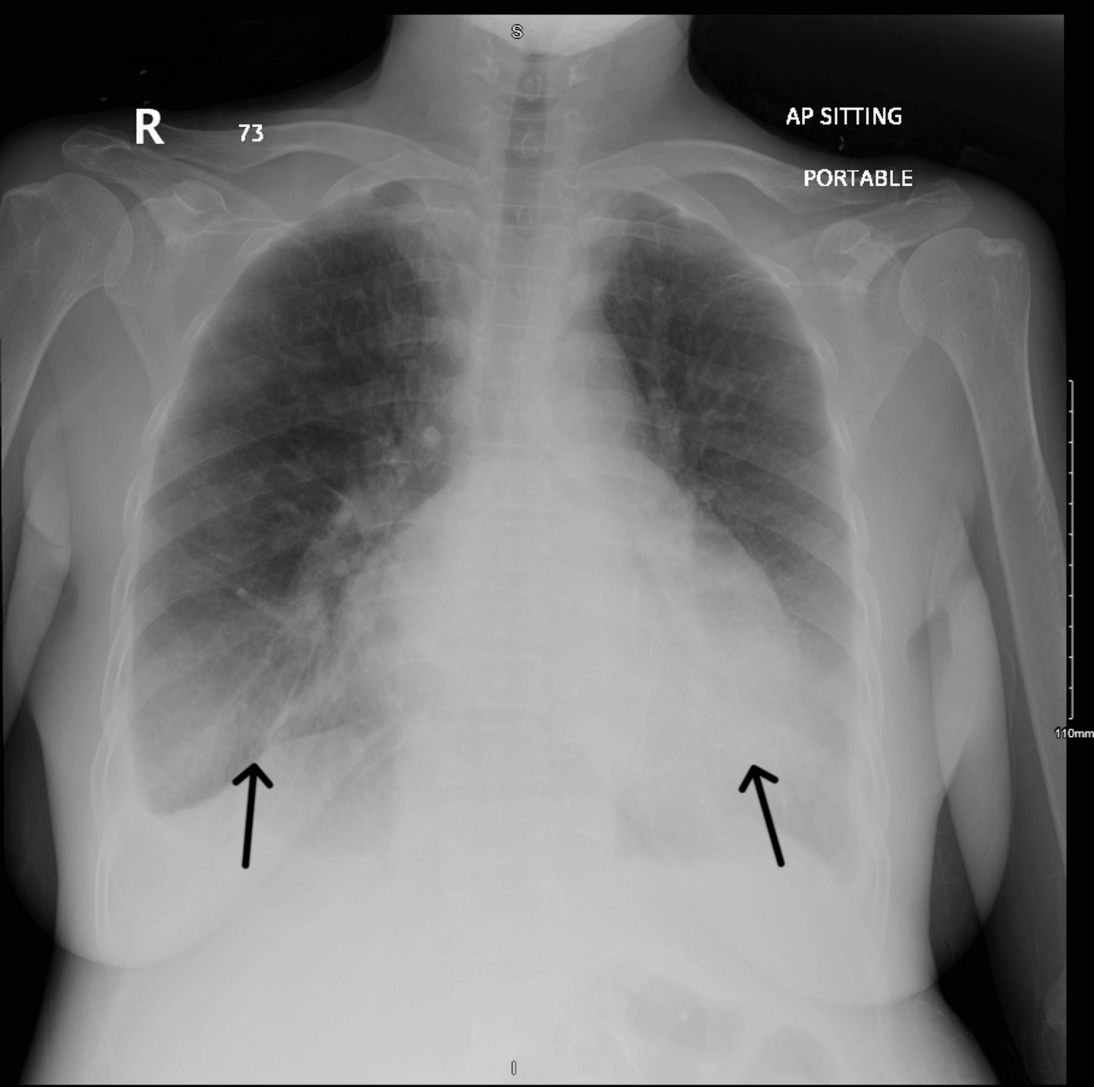
Chest X-ray showing pleural effusion (black arrows).

Her chest X-ray confirmed bilateral moderate pleural effusions, as shown in Figure 1, and abdominal ultrasound revealed moderate ascites. Both her kidneys were normal in size, with slightly increased echogenicity and mild perinephric free fluid, but without evidence of stones or hydronephrosis, on sonographic examination. Her echocardiogram showed mild concentric left ventricular hypertrophy, low-normal left ventricular systolic function with an ejection fraction of 50-55%, grade I diastolic dysfunction, and a small pericardial effusion without signs of tamponade.

A mammogram was done to rule out malignancy due to physical examination findings. The mammogram showed benign-appearing calcifications, bilateral soft tissue edema, and skin thickening, with no suspicious masses or lymph nodes observed. Furthermore, a positron emission tomography-computed tomography (PET-CT) of the whole body was performed and revealed no evidence of abnormal hypermetabolic activity suggestive of malignancy. Her further laboratory workup revealed the presence of a gamma spike and M-protein on serum protein electrophoresis. Immunoglobulin levels showed elevated IgM, decreased IgG, and normal IgA. Plasma cell dyscrasia was suspected based on the elevated IgM, presence of cryoglobulinemia, and a serum kappa/lambda free light chain ratio of 12.72.

**Figure 2:**
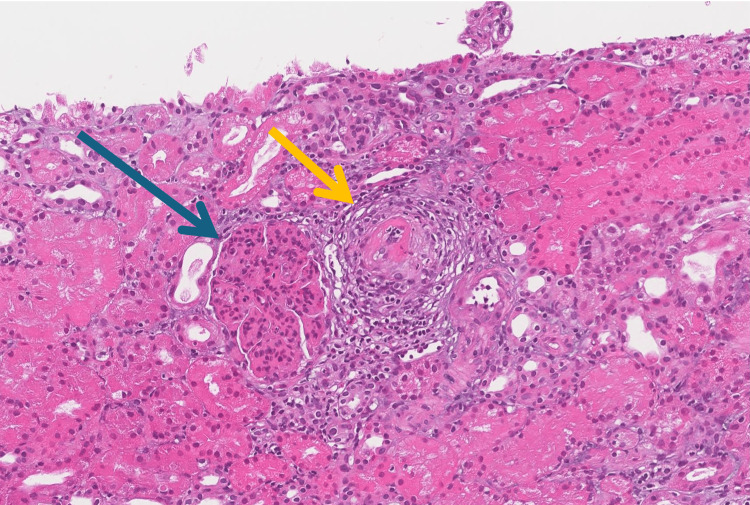
H&E section (20x) showing membranoproliferative glomerulonephritis with intra-capillary obliteration and sub-endothelial deposits (blue arrow), arterioles with cryoglobulin and vasculitis (yellow arrow).

**Figure 3:**
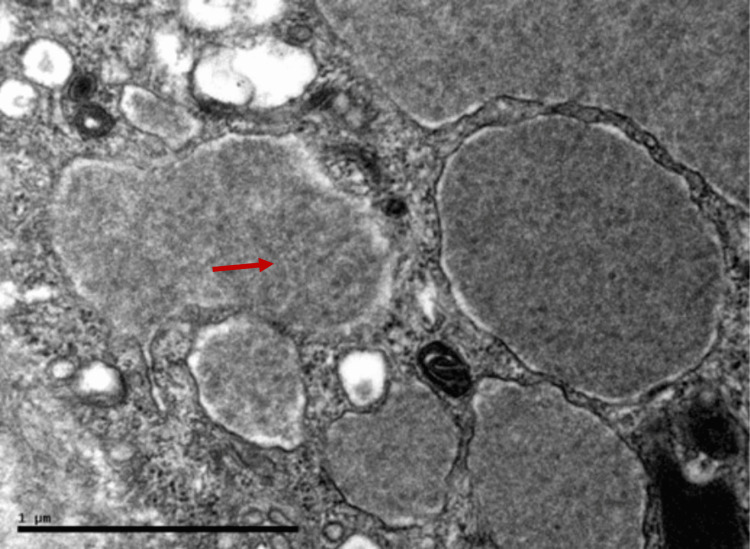
Electron microscopy shows organized intra-capillary electron-dense deposits with a microtubular arrangement consistent with cryoglobulin deposits (red arrow).

A kidney biopsy was performed, as shown in Figure 2. Light microscopy revealed features consistent with MPGN, including intracapillary obliteration and subendothelial deposits. Additionally, arterioles exhibited cryoglobulin deposition, accompanied by evidence of vasculitis. On immunofluorescence, four glomeruli were identified, demonstrating diffuse global mesangial staining for IgG and C3 (2+), along with weaker IgM staining (1+). There was also diffuse capillary wall staining for both kappa and lambda light chains (1+). No glomerular staining was observed for IgA, C4, C1q, fibrinogen, or albumin. Electron microscopy revealed large subendothelial deposits as shown in Figure 3. 

A bone marrow biopsy showed evidence of plasma cell dyscrasia, with Congo red staining negative for amyloid deposition. Flow cytometry of the bone marrow aspirate detected approximately 0.2% plasma cells positive for CD38, CD138, CD81, Beta-2 microglobulin, and cytoplasmic kappa. These plasma cells were negative for CD56, CD19, CD117, CD27, and cytoplasmic lambda. Immunohistochemistry demonstrated scattered plasma cells (5%) positively staining for CD138, kappa, and lambda with restriction toward kappa light chain.

A diagnosis of IgM myeloma versus Waldenström macroglobulinemia was initially suspected; however, bone marrow biopsy, fluorescence in situ hybridization (FISH), and cytogenetic analysis confirmed IgM kappa-related cryoglobulinemia with renal involvement, which led to her presentation with nephrotic syndrome and MPGN. She was reviewed by the hematology team, started on oral prednisolone, and received two doses of rituximab (1 g) on days 1 and 15. FISH testing revealed no chromosomal abnormalities typically associated with plasma cell dyscrasias, including negative results for IGH rearrangements, del(13q14), t(11;14), and del(17p/TP53). She exhibited an excellent response to corticosteroids and rituximab, with complete resolution of ascites, edema, and proteinuria. These findings ultimately led to the diagnosis of MGRS.

## Discussion

This case highlights an unusual presentation of IgM-kappa MGRS with type I cryoglobulinemia and nephrotic syndrome. The diagnostic approach followed a structured clinical progression: the patient presented with generalized edema, uncontrolled hypertension, and frothy urine, prompting initial labs that revealed nephrotic-range proteinuria (5.2 g/day), hypoalbuminemia, and low complement levels (C3 and C4). Suspicion of cryoglobulinemia was confirmed by positive cryoglobulin testing and elevated IgM with a markedly skewed kappa/lambda ratio. Imaging showed no malignancy or structural kidney abnormalities. The kidney biopsy demonstrated MPGN with cryoglobulin deposits and vasculitis, confirmed by light microscopy, immunofluorescence, and electron microscopy. Bone marrow biopsy revealed a plasma cell dyscrasia with kappa light chain restriction. FISH and cytogenetics ruled out myeloma-defining abnormalities, supporting a diagnosis of MGRS. Based on this evidence, the patient was treated with corticosteroids and rituximab, resulting in full resolution of proteinuria, edema, and ascites. This stepwise diagnostic and therapeutic process was essential in establishing the final diagnosis and achieving clinical improvement.

MGRS was first defined by the International Kidney and Monoclonal Gammopathy Research Group (IKMG) in 2012 and was later updated in 2017 [4]. Diagnosing this condition can be challenging due to its diverse manifestations and the difficulty in establishing a direct link between the presence of the M-protein or serum-free light chains and end-organ damage [5]. Therefore, MGRS should be considered in every patient with the presence of serum monoclonal paraproteins and evidence of kidney involvement such as changes in kidney function, hematuria, and/or proteinuria [5]. Importantly, the causal relationship in MGRS is typically assessed through kidney histology rather than hematologic evaluation alone [5]. Patients with MGRS may experience a variety of renal presentations such as proteinuria, hematuria, renal insufficiency, hypertension, or hypocomplementemia, and these are usually clustered into one of two syndromes: nephrotic or nephritic syndrome [6].

Cryoglobulinemia is the persistent presence of cryoglobulins in serum [3]. Cryoglobulins are abnormal immunoglobulins that precipitate when exposed to low temperatures and then re-dissolve when the temperature increases [3]. They have immunological and chemical features that define three types of cryoglobulinemia. Type I cryoglobulinemia, or simple cryoglobulinemia, is characterized by a single monoclonal IgG or IgM, as seen in our case [3]. Kidney involvement typically appears later in the course of the disease, with high blood pressure arising soon thereafter. Some patients may present with unusual forms of nephrotic syndrome or acute nephritic syndrome [3]. The management of cryoglobulinemia depends on its etiology, which in many cases is idiopathic or difficult to assess [3]. Consequently, the therapeutic approach to idiopathic cryoglobulinemic vasculitis remains undefined, as no research has yet established optimal treatment strategies. Nevertheless, the management of severe cryoglobulinemic vasculitis typically involves a regimen combining corticosteroids and immunosuppressants or plasmapheresis, along with the recent addition of rituximab to treatment armamentarium [7].

IgM monoclonal gammopathy is treated based on the specific condition it causes and the severity of symptoms as well as the patient’s general condition [8]. Asymptomatic cases may not require immediate treatment but are monitored regularly, while symptomatic cases like Waldenström's macroglobulinemia, cold agglutinin disease, IgM-associated peripheral neuropathy, Schnitzler syndrome, or IgM-associated amyloid light-chain amyloidosis may require intervention [8]. Treatment typically involves rituximab often combined with agents like bendamustine or bortezomib, and in some cases, steroids, interleukin-1 blockers, or ibrutinib may be used [8].

## Conclusions

The coexistence of MGRS and cryoglobulinemia in this patient, in the absence of hepatitis B and C viral serologies, supports a non-viral etiology for the disease process. The presence of an IgM monoclonal protein may play a central role in both cryoglobulin formation and kidney involvement, suggesting a possible shared underlying pathophysiology between the two conditions. However, given the individual rarity of MGRS and cryoglobulinemia, it remains possible that their simultaneous occurrence in this patient is coincidental rather than causal. This case highlights the complexity of diagnosing and managing overlapping hematologic and renal disorders and underscores the importance of considering non-viral causes when evaluating similar clinical presentations. Further research is necessary to better define the relationship between MGRS and cryoglobulinemia and to determine whether this association is a true pathogenic link or a rare coincidental finding.
